# Factors Influencing Oxidative Imbalance in Pulmonary Fibrosis: An Immunohistochemical Study

**DOI:** 10.1155/2011/421409

**Published:** 2011-05-29

**Authors:** Simona Inghilleri, Patrizia Morbini, Ilaria Campo, Michele Zorzetto, Tiberio Oggionni, Ernesto Pozzi, Maurizio Luisetti

**Affiliations:** ^1^Respiratory Disease, IRCCS Policlinico San Matteo Foundation, University of Pavia, Pavia, Italy; ^2^Department of Pathology, IRCCS Policlinico San Matteo Foundation, University of Pavia, Pavia, Italy

## Abstract

*Background*. Idiopathic Pulmonary Fibrosis (IPF) is a fatal lung disease of unknown etiology characterized by interstitial fibrosis determining irreversible distortion of pulmonary architecture. Reactive oxygen species (ROS) and markers of oxidative stress play a pivotal role in human IPF pathology, possibly through induction of epithelial-mesenchymal transition (EMT). *Methods*. We investigated by immunohistochemistry, in UIP and COP tissue samples, the expression of most relevant markers of the molecular interplay involving RAGE, oxidant/antioxidant balance regulation, tissue nitrosylation, and mediators of EMT. *Results*. In both UIP and COP, the degree of RAGE expression was similarly high, while SODs and i-NOS, diffusely present in COP endoalveolar plugs, were almost absent in UIP fibroblast foci. A lower degree of tissue nitrosilation was observed in UIP than in COP. *Conclusions*. Fibroblast lesions of UIP and of COP share a similar degree of activation of RAGE, while antioxidant enzyme expression markedly reduced in UIP.

## 1. Background

Idiopathic Pulmonary Fibrosis (IPF) is a progressive, fatal lung disease of unknown etiology characterized by alveolar wall fibrosis determining a rapid decline of lung function. The prognosis for IPF patients is poor, with a mean survival of 3–5 years [1]. Histopathologically, IPF is characterized by a specific pattern of parenchymal injury, defined usual interstitial pneumonia (UIP). The hallmarks of UIP are fibroblast foci (FF) [2], which represent both the diagnostic feature and the sites of active disease progression. They consist of parietal aggregates of activated fibroblasts and myofibroblasts which promote excessive deposition of extracellular connective matrix (ECM) in the pulmonary interstitium, thus determining the inexorable progression of fibrosis. Over the last few years, growing evidence suggested that IPF may be the result of sequential acute lung injury targeting alveolar epithelial cells and of the consequent pathological wound-healing response [3]. It has recently been proposed that fibroblasts in UIP may at least partially derive from alveolar epithelial cells through epithelial-mesenchymal transition (EMT) [4].

The presence and the role of inflammatory cells in UIP has been controversial [5]. Although early theories on IPF pathogenesis attributed to inflammatory cells a key role in the disease, a recent paradigm shift focused on different factors inducing epithelial cell damage/reparation, among which free radical-induced damage and changes in the cellular oxidant/antioxidant balance [6] have a relevant role.

Reactive oxygen species (ROS) are normal byproducts of O_2_ metabolism and have important roles in biological processes. Excess ROS, such as those produced by tobacco smoke, are recognized lung toxic factors and are associated with the development of many diseases [7]. Nitric oxide (NO°), a messenger molecule with complex biological activities, interacts with ROS producing highly reactive nitrogen intermediates. Both ROS and NO° levels in the lung are regulated by cellular enzymatic activity: NO° synthesis is catalyzed by nitric oxide synthase (NOS), in particular i-NOS. Protection from free radicals is granted by different mechanisms, among which superoxide dismutases (SODs) are the only enzymatic system decomposing superoxide radicals to H_2_O_2_. There are three different mammalian SODs: intracellular copper-zinc SOD (CuZnSOD), mitochondrial manganese SOD (MnSOD) and extracellular SOD (ECSOD) [8, 9]. SODs have been detected in all classes of lung cells but show significant variability in cell-specific localization and expression. 

It has been shown that oxidant/antioxidant imbalance can activate multiple molecular pathways that culminate in the induction of EMT in target cells: ROS activate SMAD2 and MAPK signaling which are crucial mediators of EMT [10]; on the other hand, NO° attenuates TGF-*β*1-induced EMT in alveolar epithelial cells [11].

The activation of the receptor for advanced glycation products (RAGE), a recently described member of the immunoglobulin super-family of cell surface receptors, has been shown to be involved in renal fibrogenesis through both TGF-*β*1-dependent and independent EMT activation pathways [12, 13]. Recent interest has also grown on its possible involvement in lung pathology, where RAGE is most abundant at a biochemical level [14]. So far, RAGE has been implicated in acute lung injury, and in pulmonary fibrosis in bleomycin rat models where it induces EMT in AEC [15].

**Figure 1:**
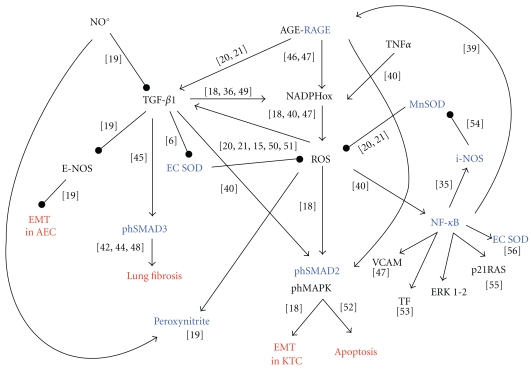
The figure shows the complex interplay involving RAGE, reactive oxygen and nitrogen species, antioxidants, profibrotic cytokines, and their signalling pathways, and, in red, the most relevant effects of pathway activation. The molecules investigated in the present study are coloured in blue. The numbers in square brackets refer to bibliography citations.

The tightly interconnected molecular pathways linking RAGE, NO, TGF-*β*1, ROS and SODs are summarized in Figure 1, with references to specific studies for each link.

The present study was aimed at analyzing in UIP tissue samples, and especially in fibroblastic foci, the expression of the most relevant markers of the complex molecular interplay involving RAGE, the enzymes regulating the oxidant/antioxidant imbalance and tissue nitrosilation, and mediators of EMT. To assess the specificity of the expression patterns observed in UIP, the results were compared with those obtained in cryptogenic organizing pneumonia (COP), an idiopathic lung disease characterized by deposition of fibroblasts and connective tissue, which however carries a favorable prognosis. We are aware that *in situ* protein expression analysis through immunohistochemistry has a number of limits: in particular the technique allows only a rough quantification of the intensity of protein expression and a suboptimal definition of cellular localization. However, immunohistochemistry remains the only method to correlate in vivo protein expression, tissue morphology and cellular phenotype, in particular in human tissue samples, where single cell-protein analyses can hardly be performed.

## 2. Materials and Methods

### 2.1. Patients

The immunohistochemical study was performed on lung samples obtained at video-assisted thoracoscopy from 42 consecutive patients between 2000 and 2007, in which the histopathological examination showed the typical morphological features of either UIP (29 patients) or COP (13 patients). The final diagnoses of UIP and COP were based on the diagnostic criteria of the American Thoracic Society/European Respiratory Society Consensus Classification System after evaluation of all clinical, laboratory, and instrumental data [2, 16]. None of the patients received treatment before surgery. The work was approved by the Institutional Ethics Committee. All patients underwent complete pulmonary function testing, measurement of carbon monoxide transfer factor (TlCO), arterial blood gases at rest and after exercise, chest X-ray and high resolution CT, and serological screening for connective tissue disease.

### 2.2. Controls

Control samples for the immunohistochemical study were obtained from normal tissue areas of 8 surgical lobectomy specimens taken from patients with lung cancer.

### 2.3. Immunohistochemistry

The immunohistochemical panel comprised antibodies and antisera specific for RAGE, MnSOD, ECSOD, i-NOS, nitrotyrosine (as an indirect index of oxidative stress), p65 subunit of activated NF-*κ*B, phosphorylated SMAD2 and SMAD3, as detailed in Table 1. Four micrometer thick paraffin sections were incubated overnight at 4°C with primary antibodies, diluted as indicated (Dako antibody diluent, DakoCytomation, Carpinteria, CA) in Table 1. Optimal pretreatments are detailed in Table 1. The reactions were revealed with the avidin-biotin-peroxidase complex (Dako LSAB+ System, Dako), using diaminobenzidine tetrahydrochloride as chromogen substrate (Dako). Each reaction set included positive controls as suggested by the manufacturer and a negative control slide exclusively incubated with the dilution buffer. All immunostained slides were examined at light microscopy by two independent observers who recorded the cell types expressing each antigen and who semiquantitatively scored the intensity of protein expression. Cell staining intensity was graded as follows: no staining (0), weak (+), moderate (++), and intense (+++). In case of disagreement, slides were reevaluated collectively to obtain a final agreement on the score.

## 3. Results

### 3.1. Epidemiologic Data

The mean age of UIP patients was 58.6 years (range 39–81); 25 of them were males and 4 females. The mean age of COP patients was 55.3 years (range 43–81); 9 of them were males and 4 females.

### 3.2. Usual Interstitial Pneumonia

**Figure 2:**
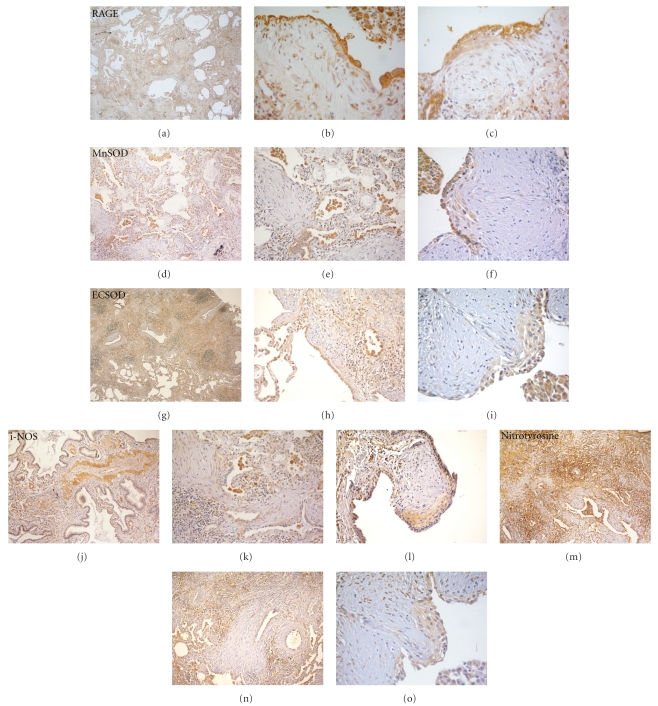
RAGE (a)–(c), MnSOD (d)–(f), ECSOD (g)–(i), i-NOS (j)–(l), and nitrotyrosine (m)–(o) expression in UIP. Low power images (a, d, g, j, m) show an overall high degree of protein expression in the lung tissue. In FF, RAGE (b) expression is observed in type II alveolar pneumocytes and stromal cells, MnSOD (e) and ECSOD (h) are expressed in epithelial cells but not in fibroblasts, i-NOS expression (k) is present in inflammatory cells and, to a lower degree, in epithelia; the overall amount of nitrotyrosine is extremely low (n). In bronchiolar basal metaplastic cells of sandwich fibroblast foci, the expression of RAGE (c), MnSOD (f), and ECSOD (i) is less strong than that in overlying alveolar pneumocytes, while i-NOS (l) and nitrotyrosine (o) stain is negative in alveolar pneumocytes, but present in metaplastic cells.

RAGE expression was globally increased in UIP lungs (Figures 2(a)–2(c)): type I and especially type II alveolar pneumocytes were strongly immunoreactive (Figure 2(b)), at the cytoplasmic and membrane level, as well as bronchial epithelia, inflammatory cells, and endothelia. In FF, both the epithelial component and stromal fibroblasts expressed RAGE; squamous metaplastic cells of FF also expressed RAGE in their cytoplasm (Figure 2(c)).

MnSOD granular, mitochondrial immunoreactivity was observed in type II alveolar pneumocyte and macrophage cytoplasms (Figures 2(d)–2(f)). In FF, surface pneumocytes showed the strongest reactivity, while MnSOD expression in squamous metaplastic cell was less intense (Figure 2(f)). Occasional fibroblasts were weakly reactive.

ECSOD was also observed in inflammatory cells, type II alveolar pneumocytes and in endothelia (Figures 2(g)–2(i)). In surface and metaplastic epithelial cells of FF the expression was less strong than in type 2 pneumocytes (Figures 2(h) and 2(i)). The stroma of FF was constantly negative.

i-NOS expression was mostly observed in medium and small vessels (Figure 2(j)). Low degree of reactivity was present in macrophages and type II alveolar epithelia (Figure 2(k)), while in FF metaplastic squamous cells appeared to be more reactive than overlying pneumocytes (Figure 2(l)). Stromal cells were faintly positive.

Nitrotyrosine expression was diffuse in UIP lungs; a stronger stain was observed in interstitial area infiltrated by larger amounts of inflammatory cells (Figure 2(m)) than in the stroma of FF (Figure 2(n)), which was almost negative. Epithelial accumulation of nitrotyrosine was also observed, both in type II pneumocytes and in metaplastic squamous cells of FF (Figure 2(o)).

**Figure 3:**
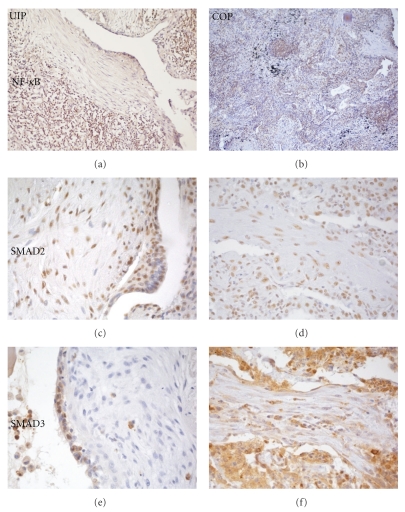
Expression of signal transduction mediators in UIP (a, c, e) and in COP (b, d, f). In both lesions, NF-*κ*B expression is only observed in infiltrating lymphocytes (a, b), while phosphorylated SMAD2 is present in a large number of epithelial, inflammatory and mesenchymal cells (c, d). SMAD3 is expressed in the nuclei of epithelial cells overlying FF in UIP (e), while diffuse cytoplasmic stain characterizes COP fibroblast plugs (f).

In UIP, nuclear expression of the p65 component of active NF-*κ*B was limited to interstitial lymphocytes and sporadic noninflammatory cells (Figure 3(a)). Nuclear SMAD2 expression was present in a large proportion of epithelial cells, alveolar type I, type II, bronchial and metaplastic squamous cells, and in fibroblasts and myofibroblasts of FF (Figure 3(c)). SMAD3 expression, on the contrary, was mostly cytoplasmic, with only scattered cells showing nuclear reactivity (Figure 3(e)). Score of immunohistochemical expression are summarized in Table 2.

### 3.3. Cryptogenic Organizing Pneumonia

**Figure 4:**
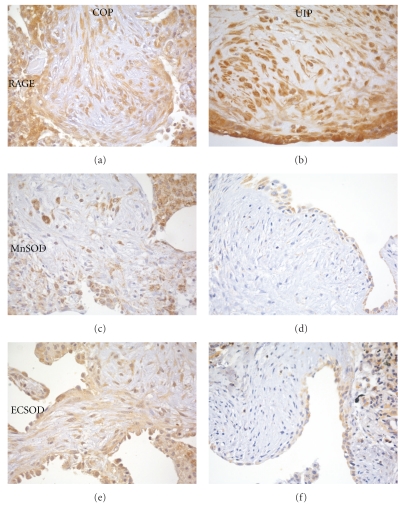
Comparison between organizing pneumonia fibroblast plugs (a, c, e) and UIP FF (b, d, f). RAGE expression is strong in both lesions, in epithelial and mesenchymal cells (a, b). MnSOD (c, d) and ECSOD (e, f) expression is stronger in COP, where it is present in epithelial and mesenchymal cells, than in UIP, where only epithelial cells are immunoreactive.

**Figure 5:**
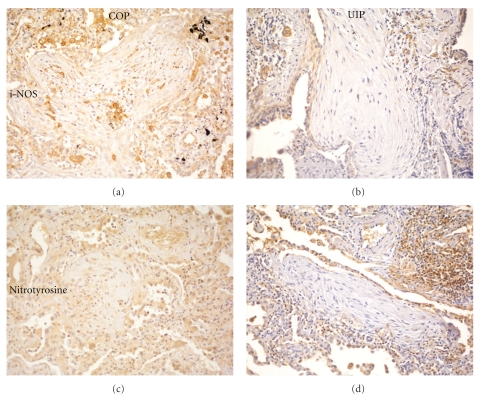
Comparison between organizing pneumonia fibroblast plugs (a, c) and UIP FF (b, d). Higher i-NOS expression is present in COP (a) than in UIP (b), mostly due to endothelial and inflammatory cell stain, but also to fibroblast expression, Epithelial cells in FF are more reactive than in COP. Nitrotyrosine reactivity is diffuse and intense in all COP cellular components (c). In UIP FF (d) nitrotyrosine expression is limited to epithelial cells and inflammatory aggregates (upper right corner), while stromal cells are completely negative.

Strong expression of RAGE was observed in hyperplastic pneumocytes and stromal fibroblasts of COP endoalveolar plugs, as well as in inflammatory cells (Figure 4(a)). The same cells were also reactive for MnSOD and ECSOD (Figures 4(c), 4(e)). Strong diffuse MnSOD reactivity was observed in the tissue due to epithelial and inflammatory cell expression; ECSOD was also expressed on the extracellular matrix. i-NOS was mostly expressed in vessels and macrophages; alveolar epithelia and stromal cells expressed lower reactivity. Stromal cells in OP fibroblastic plugs expressed higher degrees of MnSOD (Figure 4(c)) ECSOD (Figure 4(e)), and of i-NOS (Figure 5(a)) than fibroblasts of UIP FF (Figures 4(d), 4(f), and 5(b), resp.).

Nitrotyrosine deposition in the cellular and extracellular components, including the stroma of fibroblastic plugs, reflected the degree of cell activation (Figure 5(c)) and in the stroma was definitely higher than in UIP FF (Figure 5(d)). A large number of inflammatory and rare epithelial cells showed nuclear expression of the p65 component of active NF-*κ*B (Figure 3(b)). Analogously to what was observed in UIP, SMAD2 was expressed diffusely in the nuclei of epithelial, stromal, and inflammatory cells (Figure 3(d)), while SMAD3 expression was observed only at the cytoplasmic level (Figure 3(f)). See Table 2.

### 3.4. Normal Lung

MnSOD expression was limited to bronchial epithelia and macrophages; smooth muscle cells of bronchiolar walls showed some granular reactivity. Low degrees of ECSOD expression were observed in all cellular compartments and in the extracellular matrix. i-NOS was expressed in endothelia, arterial walls and macrophages. Inflammatory cells were the only site of nitrotyrosine deposition. As far as signal transduction proteins were concerned, SMAD2 nuclear expression was found in most epithelial and inflammatory cells, while SMAD3 expression was only cytoplasmic. No NF-*κ*B expression was observed in normal lung samples (Data not shown).

### 3.5. Clinicopathological Correlations

All UIP samples showed advanced fibrosis with architectural and temporal heterogeneity and FF. FF number ranged between 3 and 12 per case and did not correlate with clinical and instrumental parameters. Immunohistochemical stain results were similar in terms of cell types expressing each markers and of staining intensity within each disease group and showed no correlation with disease severity.

## 4. Discussion

In the present study; we compared the profile of expression of oxidative stress marker, major antioxidant enzymes, and mediators of ROS-induced cell activation in epithelial and stromal cells of UIP FF, which inexorably progress to irreversible lung fibrosis, and of COP mesenchymal plugs, that normally regress spontaneously or after steroid administration. The degree of RAGE expression was similarly high in both conditions, while SODs and i-NOS were diffusely present in COP, and almost absent in UIP FF. As far as nitrosative stress is concerned, lower degrees of nitrotyrosine were observed in UIP FF than in COP. Nitrotyrosine colocalized with i-NOS in both conditions. These results confirm our previous observations, that documented RAGE activation in UIP as well as in other lung pathologic conditions [17]. These observations are part of the conflicting data existing on RAGE involvement in human and experimental lung fibrosis. In contrast with our results, the pulmonary expression of RAGE and of its soluble form sRAGE resulted significantly decreased in fibrotic lungs in recent studies on bleomycin-treated animals [18, 19], suggesting a protective role for RAGE in pulmonary fibrosis. Queisser et al. [18] documented a reduced immunohistochemical expression of RAGE in human UIP as compared to controls, and suggested that the loss of RAGE in epithelial alveolar cells and fibroblasts of fibrotic lungs is associated with disturbed cellular contact and collagen adhesion, leading to fibrosis [18]. In contradiction to these data, other studies showed that RAGE knock-out mice do not develop fibrosis after bleomycin administration, and that their type 2 alveolar pneumocytes loose the capacity of undergoing EMT in response to HMGB-1 [15]. The need of further studies on RAGE function in pulmonary fibrosis is clear from the contrasting data reported and is justified by the development of RAGE inhibition as a possible therapeutic target for multiple conditions [20].

The results of the present paper are also in accordance with recent studies showing reduced ECSOD levels in UIP [6] and bleomycin animal models [21], and reduced i-NOS levels in UIP [22, 23]. In vitro studies evidenced TGF-*β*1-induced downregulation of SODs in alveolar epithelial cells [6]. The authors [6] suggested that in fibrotic lesions antioxidant downregulation reduces tissue scavenging capacity and leads to the accumulation of ROS, which in turn further induces TGF-*β*1 expression [12, 13, 24]. ROS generation in UIP FF can take place through the RAGE-NADPHox pathway, which we showed to be overexpressed both in UIP FF and in COP fibroblastic plugs. The low expression of SODs in UIP as compared to COP fibroblast lesions could contribute to explain the different behavior of the two lesions which otherwise bear a striking morphological similarity. The hypothesis of an imbalanced oxidative stress has been the rationale for the treatment of UIP patients with N-acetylcysteine, which has so far showed promising but still controversial results [25]. 

It has recently been proposed [26, 27] that bronchial basal cells play a key role in the pathogenesis of UIP lesions. These cells can be recognized morphologically and immunohistochemically in a variable proportion of FF, where they form an intermediate layer of large, squamoid cells, between the alveolar epithelia and the fibroblastic stroma, and have for this reason been defined as “sandwich foci” or squamous metaplasia. In our experiments, we noticed that epithelial cells of sandwich lesions were less reactive than type II alveolar pneumocytes for RAGE and SODs, while the degree of expression of i-NOS and nitrotyrosine was higher. Although it is difficult to say if such a slight difference of expression can be functionally significant, the interest of this observation lays in the hypothesis that these cells are an intermediate step in the epithelial-mesenchymal transition from alveolar epithelia to fibroblasts. The partial loss of proteins normally expressed in epithelial cells and the de novo acquisition of others could be a consequence of the switch from the epithelial to the mesenchymal phenotype, as documented for markers of cell adhesion and motility [27, Morbini, submitted].

We finally analyzed the most relevant signal transduction factors downstream of RAGE and TGF-*β*1 activation. The activation of RAGE increases ROS levels as a consequence of enhanced NADPH oxidase activity. ROS are recognized to mediate their signal through NF-*κ*B and phosphorylated SMAD2 nuclear transfer [10, 28]. TGF-*β*1 signaling is also mediated through SMAD2 and SMAD3 phosphorylation [29]. We unexpectedly observed ubiquitous nuclear expression of phosphorylated SMAD2 in all normal and pathological samples, while NF-*κ*B was expressed at a detectable level only in inflammatory cells. This clear discrepancy between the overexpression of RAGE in UIP fibroblasts and epithelial cells and the absence of expression of its main signal transductor, in the absence of comparable literature data, can be explained with expression levels of NF-*κ*B below the threshold of immunohistochemical detection, or with a rapid turnover that limits the amount of detectable protein. 

Nuclear expression of SMAD3, a pivotal mediator of TGF-*β*1 activation leading to fibrosis [30–32], was found in a limited number of epithelial cells in UIP fibroblastic foci, while in COP diffuse cytoplasmic stain was observed in multiple cell types. The pattern of stain was considered to be specific, since the antibody used in this study stains both cytoplasmic unbound SMAD3 and the nuclear SMAD3/SMAD4 complex. To our knowledge, SMAD2 and 3 expression has so far been documented in situ in bleomycin-induced fibrosis, and at the molecular and biochemical level in human fibroblasts, while the cellular localization of SMAD proteins in human normal and fibrotic lung has not yet been described with in situ studies. Our observation provides evidence of SMAD3 nuclear signaling in UIP lesions but not in COP fibroblast plugs and further contributes to the evidence linking the activation of the TGF-*β*1-SMAD3 axis to the development of pulmonary fibrosis in UIP. The diffuse nuclear expression of SMAD2 in normal and diseased lungs is in accordance with the hypothesis that TGF-*β*1 signaling through SMAD2 does not directly contribute to fibrosis [29, 33].

In conclusion, our study showed that fibroblast lesions of UIP and COP share a similar degree of activation of the AGE receptor and of its downstream mediators SMAD2 and NF-*κ*B, while antioxidant protein expression is higher in COP than in UIP. Moreover, SMAD3 activation appears to be specific of UIP lesions, thus confirming the direct role of TGF-*β*1 in their development. The described differences can help to understand the causes of the irreversible progression of UIP fibrosis as compared to the prompt response to steroid therapy of fibroblasts plugs of cryptogenic organizing pneumonia.

##  Conflict of Interests

The authors declare that they have no conflict of interests.

##  Authors' Contributions

I. Simona conceived the study, participated in its design and coordination, carried out the immunoassays, and drafted the paper. M. Patrizia participated in study design and coordination, established histological diagnoses, and reviewed the paper. C. Ilaria participated in study design and contributed to perform the immunoassays. Z. Michele participated in study design and contributed to perform the immunoassays. O. Tiberio participated in study design and patient selection. P. Ernesto critically revised the paper. L. Maurizio participated in its design and coordination and critically revised the paper. All authors read and approved the final paper.

## Figures and Tables

**Table 1:** Source, concentrations, and pretreatment conditions of the antibodies used in the immunohistochemical assays. MWO: microwave oven.

Name	Source	Clone	Working dilution	Pretreatment
RAGE	Abcam, Cambridge, UK	Goat polyclonal	1 : 4000	none
MnSOD	Stressgen, Victoria, Canada	Rabbit polyclonal	1 : 2000	MWO 750W 15′
ECSOD	Stressgen, Victoria, Canada	Rabbit polyclonal	1 : 400	MWO 750W 15′
i-NOS	NeoMarkers, Fremont, CA, USA	Rabbit polyclonal	1 : 200	MWO 750W 15′
Nitrotyrosin	UpState, Billerica, MA, USA	Rabbit polyclonal	1 : 800	MWO 750W 15′
NF-kB	Santa Cruz Biotechnology, CA, USA	Rabbit polyclonal	1 : 450	MWO 750W 15′
SMAD2	Chemicon, Billerica, MA, USA	Rabbit polyclonal	1 : 2000	MWO 750W 15′
SMAD3	Abcam	Rabbit polyclonal	1 : 100	MWO 750W 15′

**Table 2:** Profile and score of immunohistochemical expression in relevant lung cell types.

	Type I pn	Type II pn	Bronchiolar epithelial	UIP fibroblast foci			COP endoalveolar plugs		Macrophages
				Overlaying pneumocytes	Squamous metaplastic cells	Fibroblasts	Reactive pneumocytes	Fibroblasts	
RAGE	+	+++	+	+++	+++	+++	+++	+++	+++
MnSOD	−	+++	++	+++	++	−	+++	+	+++
ECSOD	−	+++	+	++	+	−	++	++	+++
i-NOS	−	+	−	+	++	−	+	+	+++
Nitrotyrosine	−	++	−	++	++	−	++	++	+++
NF-kB	−	−	−	−	−	−	−	−	−
Ph-SMAD2	+	+	+	+	+	+	+	+	+
SMAD3	−	+*	−	+	−	−	++*	+*	+*

*Cytoplasmic expression.

## Abbreviations

IPF: Idiopathic pulmonary fibrosis
UIP: Usual interstitial pneumonia
OP: Organizing pneumonia
FF: Fibroblastic foci
ROS: Reactive oxygen species
SOD: Superoxide dismutase
MnSOD: Manganese superoxide dismutase
ECSOD: Extracellular superoxide dismutase
i-NOS: Inducible nitric oxide synthetase
NF-*κ*B: Nuclear factor-kappa B
TGF-*β*1: Transforming growth factor beta 1
RAGE: Receptor for advanced glycation endproducts.
